# Adherence of healthcare providers to malaria case management guidelines of the formal private sector in north-western Ethiopia: an implication for malaria control and elimination

**DOI:** 10.1186/s12936-022-04379-0

**Published:** 2022-11-21

**Authors:** Mesele Damte Argaw, Thandisizwe Redford Mavundla, Kassa Daka Gidebo, Binyam Fekadu Desta, Heran Demissie Damte, Wondwosen Mebratu, Wasihun Edossa, Dereje Dillu, Aychiluhim Damtew Mitiku, Alebel Yaregal Desale

**Affiliations:** 1 USAID Transform: Primary Health Care Activity, JSI Research & Training Institute, Inc., in Ethiopia, P.O. Box 1392 code 1110, Addis Ababa, Ethiopia; 2 Department of Nursing Education, University of Witwatersrand, Johannesburg, South Africa; 3 Department of Health Studies, University of South Africa (UNISA), Pretoria, South Africa; 4 School of Public Health, Wolaita Sodo University, College of Health Sciences and Medicine, Wolaita Sodo, Ethiopia; 5 School of Public Health, Wollo University, College of Health Sciences and Medicine, Dessie, Ethiopia; 6 Department of Global Health and Health Policy, Addis Continental Institute of Public Health, Addis Ababa, Ethiopia; 7 Federal Ministry of Health, Addis Ababa, Ethiopia; 8 Emory University, Addis Ababa, Ethiopia

**Keywords:** Adherence, Malaria control and elimination, Formal private sector, Ethiopia

## Abstract

**Background:**

Malaria is an infectious disease which has been globally targeted for elimination in at least 35 of 90 endemic countries by 2030. Most successful malaria elimination country programmes have engaged the private health sector in an effort to identify, document, investigate, provide effective treatment, and follow-up cases. However, there has been limited rigorous research showing evidence of adherence among healthcare providers of the formal private health sector to national malaria diagnosis and treatment guidelines in Ethiopia, starting from malaria control to elimination phases. The aims of this study were to investigate and explain the level of adherence to malaria diagnosis and treatment guidelines among healthcare providers working in formal private health facilities in north-western Ethiopia.

**Methods:**

An explanatory sequential mixed method design was conducted in the West Gojjam Zone of Ethiopia. Quantitative data were extracted from 1650 medical records of adult uncomplicated malaria outpatients served in 11 private-for-profit health facilities. In addition, using a qualitative approach, 33 in-depth interviews (IDIs) with healthcare providers were conducted. All interviews were audio-recorded, transcribed verbatim, and analysed using eight steps.

**Results:**

Of 1650 suspected malaria cases in adult outpatients, 80.6% (1330/1650) were screen tested using microscopy and the remainder 19.4% (320/1650) were tested using multispecies rapid diagnosis tests (RDTs). Hence, the results revealed that private healthcare providers universally adhered to diagnosis guidelines. In addition, after following-up and excluding other causes of fever, 4.1% (56/1376) patients were clinically diagnosed with uncomplicated malaria. Despite this, the proportion of private healthcare provider adherence with confirmed malaria case treatment guidelines was 20.9% (69/330). In addition, 1320 (95.9%) of adult outpatients with negative laboratory results were not treated. Some of the identified determinant factors for sub-optimal adherence of healthcare providers to malaria guidelines were interruptions in supply and lack of availability of recommended anti-malarial drugs, lack of availability of quality assured laboratory supplies, and poor knowledge of the recommendations of the national standards.

**Conclusions:**

Private healthcare providers adhered to universal parasitological diagnosis, providing comprehensive counseling, and linking patients with community health workers. In addition, almost all laboratory negative patients were not treated with anti-malarial drugs. However, only one-fifth of confirmed patients were treated in line with national guideline recommendations. Malaria control and elimination efforts across Ethiopia could be improved through establishing a collaborative function of a win-win public private mix partnership model. In addition, including the data of the private health sector in the health information system could show real malaria burden and use the information to improve the adherence to malaria diagnosis, treatment, and reporting standards within the targeted era of elimination. Therefore, building the capacity of private healthcare providers and ensuring the availability of all nationally recommended drugs and supplies in private health sector facilities is recommended to improve the quality of services.

**Supplementary Information:**

The online version contains supplementary material available at 10.1186/s12936-022-04379-0.

## Background

Malaria is a serious, infectious, and life-threatening disease caused by five *Plasmodium* parasites that are transmitted to human beings through the bite of infected female *Anopheles* mosquitoes [1]. However, two of the five *Plasmodium* species, namely *Plasmodium falciparum* and *Plasmodium vivax,* pose a greater risk of morbidity and mortality in sub-Saharan Africa (SSA) [1]. In 2020, there were an estimated 241 million cases of malaria and 627,000 deaths from malaria, worldwide. The majority, 95.0% and 96.0% of malaria cases and deaths occurred in 29 countries within the SSA region [1].

During the preceding seven decades, the World Health Organization (WHO) recognized 40 countries and territories for achieving malaria-free status [2]. In addition, after 15 years of implementing the Millennium Development Goal (MDG) strategic initiatives, (2000 and 2015), the world has witnessed a reduction in malaria by 41 percent in case incidence and 62 percent in mortality rates [3]. The WHO, encouraged by this progress, has set an ambitious target for malaria elimination in at least 35 out of 90 endemic countries by the year 2030 [3].

Despite this positive progress, malaria continues to be a major public health concern in many parts of the world. Lessons from successful malaria control and elimination programmes have revealed that countries had implemented multi-pronged strategies [4]. Based on evidence-based programming exercises [5, 6], the Ethiopian Ministry of Health developed national malaria guidelines with several strategic solutions including: (1) community engagement; (2) providing prompt malaria diagnosis and timely effective treatment services at the community level, and in public and private health facilities; (3) vector controls; (4) early detection and mitigation of outbreaks; and (5) strengthening surveillance and capacitating healthcare providers [7, 8]. In the coming decade, Ethiopia intends to eliminate malaria through a stepwise approach that targets interventions in historically low transmission districts (i.e., five malaria cases per 1,000 people, per year) which will shrink its malaria maps through a progressive enrolment of whole districts, leading to a malaria free country by 2030 [9].

The national malaria diagnosis and treatment guidelines (2017) recommend a universal parasitological diagnosis using microscopy or multispecies RDTs [7, 8]. It recommends artemether-lumefantrine (AL) plus single low dose primaquine, without testing for glucose-6-phosphate dehydrogenase deficiency (G6PDd), as a first-line of treatment for uncomplicated *P. falciparum* malaria in adult populations to be implemented in the whole country. The recommended treatment for *P. vivax* malaria infection is either only chloroquine in high transmission areas or after ruling out G6PD using rapid tests, and chloroquine plus primaquine for 14 days observed treatment in malaria elimination targeted districts. Pregnant and lactating women should be treated with oral chloroquine or quinine. Furthermore, AL plus single low dose primaquine is recommended to treat presumed malaria cases. However, healthcare providers should consider the presumed diagnosis in the absence of parasitological confirmation or after all other possible causes of fever have been ruled out [7, 8] (Additional file 1).

In many African countries, adherence of private healthcare providers to their respective national malaria guidelines were found to be suboptimal [10–16]. A study conducted in eastern Uganda has documented a 50.7% (95% CI 21.2, 79.7) adherence to recommended malaria management in private-for-profit health facilities [16]. Similarly, in Nigeria, Mokuolu et al. [13] estimated a 73.8% (95% CI 71.7–75.7%) and 80.9% (95% CI 78.7–83%) adherence of private healthcare providers to malaria diagnosis and treatment recommendations. Furthermore, in Angola, Rowe et al. [17] report as low as 30.7% and 49.0% level of adherence of healthcare providers to malaria diagnosis and treatment recommendations, respectively.

In Ethiopia, free malaria diagnosis and treatment services are available at the community health programme level and in public health facilities [7, 8]. However, one-third of mothers first sought services from private health facilities for their feverish children. To improve the quality of care, since 2012, public–private mix (PPM) for malaria services were implemented in 264 private-for-profit and private-for-non-profit health facilities. In spite of that, there is limited evidence on adherence to and on the opinions of private healthcare providers on malaria diagnosis and treatment guidelines [15]. Therefore, the aims of this study were to determine and explain the level of adherence to malaria diagnosis and treatment guidelines among healthcare providers working in formal private health facilities in north-western Ethiopia.

## Methods

### Study design

An explanatory sequential mixed-method study was conducted between October 2016 and January 2017 in West Gojjam Zone, located in north-western Ethiopia [15, 18]. First, quantitative data were collected from 1,650 medical records of malaria suspected adult patients observed in 11 formal private health facilities. The second phase involved qualitative data collection using in-depth interviews (IDIs) of 33 healthcare providers. The implemented quantitative and qualitative methods are described in detail elsewhere [15, 18].

### Study setting

Ethiopia is located in the Horn of Africa with an estimated population of 115 million. Three-fourths of the 1.1 million square kilometres of landmass where two-thirds of the population live are at risk of acquiring malaria. Seasonal migrant workers spent about 3–6 months in the high malaria transmission areas and returned back home to the study areas [19]. The study was conducted in West Gojjam Zone, of Amhara Region, north-western Ethiopia. West Gojjam is one of the ten administrative zones and is located at latitude and longitude of 11° 09′ 60.00" N, and 37° 14′ 60.00" E with an average elevation of 2,466 m above sea level. Based on the projected Ethiopian 2007 national census, the estimated population of the zone for the year 2016 was 2,474,254 [20]. Health services are offered through the available health facilities which include 363 health posts, 90 health centres, and 76 private health facilities. Twenty formal private-for-profit health facilities were engaged in public–private partnerships for malaria services [20]. Malaria is endemic in the zone and contributed one-third of the malaria burden among the ten zones in the Amhara Region [21]. In addition, four of the fourteen targeted districts, namely: Finote Selam, Jabih Tehina, Bure, and Wemberma accounted for half (51,060/104,202) of all reported malaria cases in the year 2016 [21].

### Quantitative: population, sampling, and sample size

Eleven private formal medium clinics which were providing outpatient malaria case management in Finote Selam, Jabih Tehina, Bure, and Womberma districts, were categorized into two. The categorization was based on engagement in public–private partnerships for malaria services. The sample size was determined using Aga Khan Foundation’s ‘rule of thumb’ recommendation for quality assessment [22]. All eleven medium clinics were enrolled in the study. Medical records of patients served over three months (Oct–Dec 2016) were reviewed. Of 11,786 outpatients, the records of 1,650 malaria suspected patients were extracted.

### Quantitative data collection and management

The quantitative data were extracted using a pretested tool. The questionnaire was prepared based on identified variables in national malaria guidelines [8]. The structured questionnaires were designed to capture the socio-demographic characteristics of patients such as age, sex, weight, and place of residence. In addition, chief complaints and the history of present illness were reviewed for malaria. Furthermore, data documented in patient charts on malaria diagnosis method, parasite load, and prescribed and administered anti-malaria drugs were extracted. Sixty-one patient records were dropped due to observed signs and symptoms of severe and complicated malaria. The questionnaire was developed in the English language and pretested in private health facilities located in two districts of the same zone within the study area.

### Adherence determination

The adherence of healthcare providers to the malaria diagnosis and treatment practices was determined based on the recommendations of national malaria guidelines. Healthcare providers who adhered to national guidelines diagnosed malaria using microscopy or multispecies malaria RDTs. Similarly, adherence of healthcare providers was evaluated according to recommendations for presumed malaria, *P. vivax*, *P. falciparum*, and mixed infections for high transmissions. Extracted data were categorized by a team consisting of a medical doctor, public health officers, laboratory technicians, and nurses. Malaria diagnoses were categorized as adhered to or not adhered to using the universal parasitological confirmation. Then, the diagnosis methods were evaluated as presumed or confirmed malaria cases. The species of confirmed malaria cases were further assessed and documented as “*P. falciparum*”, “*P. vivax*”, and “mixed infection (P*. falciparum* + *P. vivax*)”, using microscopy or RDTs.

### Quantitative data analysis

The data were entered, cleaned, and analysed using the statistical package for social science research version 25 for Windows (SPSS, Chicago, IL, USA). The results of the descriptive statistics are presented using frequency tables. Continuous variables are expressed as mean ± standard. Where appropriate, the Chi-square (*X*^2^) test was employed to compare distribution of categorical variables or groups, when less than 20% of cells have the expected frequencies < 5. However, when the samples were small or more than 20% of cells had expected frequencies < 5, Fisher’s exact test was conducted. To claim the existence of statistical differences between groups or categorical variables, the selected critical value was p < 0.05 [23, 24]

### Qualitative: population and sampling

The qualitative inquiry targeted formal private healthcare providers including medical doctors, public health officers, nurses, and laboratory technicians who provide outpatient malaria care services in the study area.

### Qualitative data collection and management

A semi-structured IDI guide was developed in English and translated into Amharic⁠—the local official language of the study area. The interviews were facilitated in Amharic. Each IDI took from 45 to 60 min. All interviews were audio-recorded, transcribed verbatim, and handwritten notes were taken by the data collectors. The data collection ceased upon saturation of 33 in-depth individual interviews [25].

### Qualitative data analysis

Qualitative content analysis was implemented in eight steps using transcripts of interviews and handwritten notes [25]. Firstly, all transcripts and handwritten notes were gathered. Secondly, two investigators read and re-read sample files and identified words, phrases, or sentences related to adherence to malaria diagnosis and treatment guidelines. Thirdly, the investigators developed categories for codes using a deductive approach, taking the guidelines as an analysis framework. The fourth step was holding discussions on and validation of codes and categories to ensure consistency between investigators. The fifth step involved coding all files and text. The sixth step was checking the consistency of the use of codes, categories, and themes. The seventh step was interpreting the themes and inferring the findings based on observed patterns, relationships, and properties of codes, categories, and themes. The eighth step was presenting the results with supporting quotes and verbatims.

Furthermore, the raw data were recorded by experienced qualitative researchers and consensus discussions were arranged to define themes. During data analysis, a single theme, two categories, and six subcategories were identified [26].

### Measures for ensuring trustworthiness

To address issues of trustworthiness, the paradigms of Guba (1985) [27] were followed. The four proposed parameters are credibility, transferability, dependability, and confirmability [28, 29]. The credibility of this research was maintained through development and presentation of the implemented approaches in the methods section. In addition, the investigators applied a triangulation of data and spent more than three months in the study area. The transferability of this research was ensured through a thick description of the methods section and the results are presented with associated quotes. The dependability of the study was maintained with independent coding of the text and reaching of a consensus between two investigators. The confirmability of this study was ascertained using triangulation of collected data from medical records [28, 29].

### Ethical considerations

The study was approved by the institutional review boards (IRB) of the University of South Africa (UNISA) and the Amhara Regional State Health Bureau, Research and Technology Transfer Core Process. A supporting letter was obtained from the West Gojjam zone health department and Finote Selam, Jabih Tehina, Bure, and Wonberma district health offices. Additionally, all participants were informed and signed consent forms to participate in the IDIs. To maintain the confidentiality of collected data, anonymity was maintained throughout the research process [28, 29].

## Results

The results of this study are presented firstly through quantitative study findings including demographic characteristics of adult malaria outpatients and adherence of private healthcare providers to national malaria diagnosis and treatment guidelines. This is followed by qualitative IDI findings.

### Quantitative study findings

Table 1 presents the socio-demographic characteristics of 1,650 malaria suspected outpatient service beneficiaries in 11 formal private health facilities located in West Gojjam Zone of north-western Ethiopia. The mean age [standard deviation (± SD)] of the study’s outpatient participants was 30.2 (± 12.4) years. Slightly higher than half (57.6%) of patients were male. In terms of residence, 55.2% of patients were urban residents. The age distribution of patients is much higher in PPM partnership health facilities than non-partner health facilities at *X*^2^ = 14.29, *p* = 0.006.

**Table 1:** Socio-demographic characteristics of malaria suspected outpatients in 11 private health facilities, Oct 2016–Jan 2017

Characteristics	Responses	PPM facilities		Non-PPM facilities		Chi-square	P-value
		Frequency	%	Frequency	%		
Age in years	18–20	227	25.0%	219	29.5%	14.29	0.006
	21–30	354	39.0%	316	42.6%		
	31–40	145	16.0%	94	12.7%		
	41–50	109	12.0%	76	10.2%		
	51 +	73	8.0%	37	5.0%		
	Mean (± SD): 30.2 (± 12.4); Median: 28; Range: 63 (81–18)						
Gender	Male	545	60.0%	405	54.6%	4.94	0.026
	Female	363	40.0%	337	45.4%		
Place of residence	Urban	505	55.6%	405	54.6%	0.176	0.674
	Rural	403	44.4%	337	45.4%		

### Adherence to malaria diagnosis guidelines

One thousand six hundred and fifty malaria suspected cases who had a fever or history of a fever for 48 h at the time of the examinations were clinically identified. All patients were referred for laboratory services to investigate and confirm their diagnosis. Of these, 55.0% (908) and 45.0% (742) of patients were screened in PPM and non-PPM health facilities, respectively. Most patients 80.6% (1330/1650) were screened using blood film through microscopy. The remaining 19.4% (320/1650) were screened for malaria using multispecies RDTs. The malaria parasite positivity rate was 16.6% (274/1,650). In addition, among the malaria parasite negative patients, 4.1% (56/1376) were clinically reassessed and diagnosed as presumed malaria cases (Table 2).

**Table 2:** Adherence of healthcare providers to national malaria guidelines, Oct 2016—Jan 2017

Characteristics	PPM facilities		Non-PPM facilities		Chi-square	P-value
	Frequency	%	Frequency	%		
Diagnosis methods						
Microscopy n1 = 1330	150	83%	72	48%	45.74	0.001
Multi-species RDTs n2 = 320	16	9%	36	24%		
Presumed malaria n3 = 1,376	14	8%	42	28%		
Parasitological confirmed malaria						
* P. falciparum*	92	51.1%	52	46.8%	1.45	0.483
* P. vivax*	46	25.6%	36	32.4%		
Mixed (P. f or both P. f and P. v)	28	15.6%	20	18.0%		
Treatments adhered to guidelines						
*P. falciparum*, mixed (P. f or both P. f and P. v) & presumed malaria						
AL plus primaquine (sld)	0	0.00%	0	0.00%	NA	NA
Quinine plus primaquine (sld)	0	0.00%	0	0.00%		
*P. vivax*						
Chloroquine	39	84.8%	29	80.56%	0.255	0.614
Treatments not adhered to guidelines						
*P. falciparum*						
AL or quinine	82	89.1%	12	23.1%	83.31	0.001
AL plus artemether injection and antibiotics	7	7.6%	0	0.0%		
Artemether injection	3	3.3%	40	76.9%		
Mixed (P.f or both P. f and P. v)						
AL or quinine	10	35.7%	2	10.0%	14.1	0.001
AL plus artemether injection and antibiotics	18	64.3%	8	40.0%		
Artemether injection	0	0.0%	10	50.0%		
*P. vivax*						
AL or chloroquine plus primaquine	3	6.5%	0	0.0%		*1.000
Chloroquine plus artemether injection and antibiotics	2	4.3%	4	11.1%		
Artemether injection	2	4.3%	3	8.3%		
Presumed malaria						
AL or quinine	4	28.6%	18	42.9%		*0.756
Artemether injection and antibiotics	1	7.1%	1	2.4%		
Chloroquine	9	64.3%	23	54.8%		

NB *Fisher exact test statistic value

### Malaria treatment for *Plasmodium vivax* infections

The majority, 39 (84.4%) and 29 (80.5%) of *P. vivax* malaria patients were treated with first-line recommended guidelines in PPM and non-PPM facilities, respectively. These treatment practices do not show a statistically significant difference by partnership status at *X*^2^ = 0.255, *p* = 0.614.

### Malaria treatment for *Plasmodium falciparum* or mixed infections

In both PPM and non-PPM private health facilities, no single confirmed *P. falciparum*, or mixed parasite patient was treated with first-line or second-line treatment guidelines. Though the treatment was not in line with the guideline, in PPM facilities, 82 (89.1%) of patients with *P. falciparum* were treated with AL or quinine, while 12 (23.1%) of patients in non-PPM facilities were treated with AL or quinine.

### Malaria treatment for presumed malaria cases

Out of 56 presumed malaria cases, 4 (28.6%) and 18 (42.9%) of patients were treated in line with the guidelines in PPM and non-PPM health facilities, respectively. Nine (64.3%) and 23 (54.8%) of patients were treated with oral chloroquine which was against the national malaria treatment guideline recommendations in PPM and Non-PPM health facilities, respectively.

### Qualitative study findings

#### Demographic characteristics

Among 33 healthcare providers who participated in the IDIs, 23 (69.7%) were male. The mean age with standard deviation (± SD) of the IDI participants was 33.4 (± 8.3) years, median age was 32 years, and the age range of the participants was 35 (23–58) years. The professions of the IDI participants were nurses (11), public health officers (10), laboratory technicians (10), and medical doctors (2). The mean service year tenure with standard deviation (± SD) of the study participants was 10.3 (± 8.9) years, the median service year was eight years, and the range of service years was 36 (37–1) years (Table 3).

**Table 3:** Demographics of healthcare providers

Variables	Health care providers (n = 33)
Gender	
Male	69.69% (23/33)
Female	30.03% (10/33)
Age (in years)	
Range	35
Median	32
Mean	33
Marital status	
Single	30.3% (10/33)
Married	69.7% (23/33)
Education	
College (diploma /10 + 3)	60.7% (20/33)
Bachelor’s degree (12 + 4)	33.3(11/33)
Master’s degree (12 + 6)	6.0% (2/33)
Profession	
Nurse	33.3% (11/33)
Laboratory technician	30.3% (10/33)
Public health officer	30.3% (10/33)
Medical doctor	6.1% (2/33)

#### Adherence of healthcare providers to national malaria case management guidelines

This study dealt with the adherence of healthcare providers to case management recommendations of the Ethiopian national malaria guidelines. The IDI participants’ adherence was explored. Two categories and five subcategories were identified within this study (Table 4).

**Table 4:** Theme, categories and subcategories identified in IDIs

Theme	Categories	Subcategories
Adherence of healthcare providers to national malaria case management guidelines	Adherence to malaria diagnosis standards	Microscopic diagnosis Multispecies malaria RDT diagnosis Clinical or Presumed diagnosis
	Adherence to malaria treatment standards	Antimalarial prescription and dosing Administration of first dose antimalarial treatment (direct observed therapy) Comprehensive counselling Close follow-up

#### Category 1.1: Adherence of healthcare providers to malaria diagnosis standards

This category emerged from three sub-categories, namely: (1) microscopic diagnosis; (2) multispecies malaria RDTs; and (3) clinical /presumed diagnosis. Detailed descriptions and related verbatims are presented below.

##### Sub-category 1.1.1: Microscopic diagnosis

The participants explained that for all febrile patients, malaria was top on the list of diseases of their differential diagnoses. Suspected patients were sent to laboratory units for confirmation of the diagnosis through microscopic investigation for blood parasites. The following statement was common among many healthcare providers:

Almost all feverish patients are tested for blood parasites. The most common and confirmed diagnosis using microscopy is the malaria parasite.

##### Sub-category 1.1.2: Multispecies malaria RDTs diagnosis

A few participants stated that due to the unreliable quality of laboratory supplies available in the market, they preferred to screen malaria suspected cases using multispecies malaria RDT kits. The following statement was given by a healthcare provider:

…the quality of laboratory reagents is unreliable; I would prefer to diagnose malaria using multispecies RDT kits…

##### Sub-category 1.1.3: Presumed malaria diagnosis

Some of the healthcare providers reported that they reassessed patients with negative laboratory test results before resuming treatment. The following statements were made by two healthcare providers:

…we are very familiar with the signs and symptoms of malaria as most patients have fever or history of fever for at least 48 h. The other indicator is a patient who lives in a malaria endemic area or who has had a history of travel to a malarious area within the previous 30 days. Once they fulfill these criteria and show negative laboratory results, we would investigate other causes of fever and continue our follow-up. Then, we treat malaria in a few cases, based on our clinical judgments… [IDI: HF8, HW2].

Since we don’t have quality assured laboratory services, we sometimes doubt the proficiency of the laboratory professionals. It is difficult for us to rule out malaria with negative laboratory reports. We would prefer to over-treat patients for malaria. [IDI: HF2, HW1].

#### Category 1.2: Adherence of healthcare providers to malaria treatment standards

Under this category, the researchers identified three subcategories, namely: (1) anti-malarial prescription and dosing; (2) first dose anti-malarial administration; and (3) comprehensive counseling and follow-up services. These subcategories are discussed below.

##### Subcategory 1.2.1: Anti-malarial prescription and dosing

In this study, half of the participants followed the proper treatment recommendations for uncomplicated *P. vivax* malaria cases. The healthcare providers properly prescribed the correct dose of anti-malarial drugs for malaria patients. The following verbatim quotation shows the treatment of *P. vivax* malaria patients by the majority of healthcare providers:

P. vivax malaria is treated with chloroquine 25 mg/kg body weight in three divided doses.

…we treat P. vivax adult malaria cases with ten chloroquine tablets… [IDI: HF2, HW1]

Nonetheless, the adherence of some of the participants to the standard anti-malarial drugs was suboptimal.

…we used to treat P. vivax malaria cases with quinine 600 mg three times per day for seven to ten days…sometimes, we also supplement chloroquine with tetracycline or doxycycline or clarithromycin or clindamycin. [IDI: HF8, HW1].

A health worker also described the reason for the non-adherence with national treatment guidelines as follows*:*

I use all possible means to cure my patients. I treat my patients with three or four anti-malarial drugs. If a patient is identified with P. falciparum malaria, I prescribe AL and doxycycline or clarithromycin.…it is also in line with WHO guidelines. This is because as this is a private-for-profit firm, if I fail to cure my patients even once no one would visit us again. [IDI: HF7, HW2].

##### Subcategory 1.2.2: Administering anti-malarial drugs under healthcare provider supervision (direct observed therapy: DOT)

In this study, most participants reported that their patients took the first dose of the anti-malarial drugs under their supervision. The following statement was made by the healthcare provider from HF3:

My clinic is working with a public–private mix partnership for malaria services. All patients diagnosed with malaria take their first dose of anti-malarial drugs under my supervision and I monitor my patients for half an hour. If the patient vomits, I will facilitate a re-administration of the first dose of anti-malarial drugs. [IDI: HF3, HW3].

However, in a little less than half (5/11) of the targeted private health facilities where no anti-malarial drugs were stocked, the healthcare providers reported that there are patients who do not return to the health facilities to take the first dose under their supervision. An individual in-depth interviewee from HF9 had this to say:

It is not legal for us to hold anti-malarial drugs within this facility, so we send our patients to rural drug vendors with a prescription. Some patients don’t return. It is difficult to exercise DOT in our setup. [IDI: HF9, HW1].

##### Subcategory 1.2.3: Comprehensive counseling

The majority of the IDI participants reported that they used to facilitate patient counseling sessions. The areas they used to adequately deal with adult malaria patients include its causes, mode of transmission, prevention strategies, doses, and schedules of drugs. In addition, they discuss with patients when to return to health facilities and the importance of compliance with anti-malarial drugs. The health workers also reported that they would ask patients to recount health messages they received during the two-way discussion sessions. The following statements was made by an IDI participant in HF1:

…the counseling session addresses several things. I firmly tell the patient that they are suffering from malaria. Then, I tell the patient malaria is transmitted through the bite of an infected female Anopheles mosquito. Following a brief explanation of the cause, mode of transmission, and prevention methods, I tell patients the dose and schedules of the prescribed anti-malarial drugs. The other important health messages addressed during the counseling sessions are indications and contraindications of when to return to a health facility. Finally, before the session ends, I ask patients to recount what we have discussed and answer questions. [IDI: HF1, HW3].

Two healthcare providers from HF2 and HF11 also stated that they offered more focused counseling services on the recommended foods and fluids, and when to return to health facilities:

We advise patients to consume more fluids and food. In particular, patients who take AL are encouraged to consume milk and fatty meals. [IDI: HF2, HW3]

I encourage customers to return anytime if the condition worsens or if the fever persists for more than 48 h. [IDI: HF11, HW2]

##### Subcategory 1.2.4: Close follow-up

Most participants stated that they used to link malaria patients with the community health system. Community health workers are informed of the patient’s diagnosis as well as the dose and schedule of dispensed anti-malarial drugs. The following statement from a healthcare provider clearly describes the linkage of malaria patients from a private health facility to the public health system.

I used to link malaria patients with health extension workers for close follow-up. With the consent of patients, I informed the health workers about the dose and schedule of dispensed anti-malarial drugs to encourage them to adhere to our advice. In addition, community health workers are also expected to scan nearby households for any case build-up. [IDI: HF3, HW3].

## Discussion

The WHO highlighted the importance of adherence to its recommendations on malaria diagnosis and treatment guidelines in all malaria control, pre-elimination, and elimination programmes. Based on the global guidance, Ethiopia’s malaria control and elimination programme has formulated an early accurate diagnosis and effective treatment using community health programmes and public and private health facilities [5, 7]. In addition, providing a standardized service is an entry point to ensuring the quality of the malaria case management and adherence of healthcare providers with national guidelines contributes significantly to the prevention of the spread of the disease and occurrence of drug resistance parasites [7, 8]. However, during the last two decades, a small number of private-for-profit and private-for-non-profit health facilities were engaged in malaria services through a formal partnership with the public health sector in Ethiopia. This explanatory mixed-method study aimed to determine and investigate the determinants of the adherence of private healthcare providers working in PPM and non-PPM health facilities to national malaria guidelines in north-western Ethiopia.

The findings of this study revealed that all malaria suspected patients were investigated for parasitological confirmation using microscopy (80.6%) and multispecies RDTs (19.4%). In addition, the healthcare providers adhered to national guideline recommendations on avoiding treatment for 95.9% of outpatients with negative laboratory test results, provision of comprehensive counseling, administering anti-malarial drugs under supervision, and ensuring continuity of care through follow-up of patients by linking malaria patients with community health workers. This finding was in line with Namuyinga, et al. [30] who report an excellent level of adherence of healthcare providers to universal test and treatment guidelines in Malawi.

But no single *P. falciparum* or mixed (*P. falciparum* and *P. vivax*) malaria patient was treated in line with the first line recommendation of the malaria guidelines. The study documented suboptimal adherence of healthcare providers to treatment guidelines in both PPM and non-PPM health facilities. This is similar to poor adherence to guidelines by health workers in Angola in treating one-third up to half of uncomplicated malaria patients [17]. Therefore, these results can inform policy makers, programme managers, and healthcare providers through identifying areas for improvement for future successful implementation of phase-based malaria elimination initiatives in Ethiopia.

The qualitative data analysis revealed the varied gaps in adherence to national guidelines i.e., diagnosis, treatment of *P. vivax*, and treatment of *P. falciparum.* These findings were explained by qualitative data. The private sector is expected to provide diagnosis services and ensure availability of laboratory services which indicate that good quality of care is being provided for communities. In Ethiopia, *P. vivax* malaria has long been treated with chloroquine and the revised guidelines maintain this recommendation in high transmission areas [7]. Despite this, unless there is a public private mix partnership model in place, the private sector should provide treatment of malaria patients with prescriptions to drug vendors or stores. Furthermore, the healthcare providers lack orientation on updated guideline recommendations in regards to a nationwide integration of sld primaquine with AL for *P. falciparum* or mixed malaria cases [8].

Healthcare providers working in PPM facilities were more likely to adhere to national guideline recommendations than their counterparts working in non-PPM facilities. This finding was in line with previous studies where PPM facilities had improved malaria case management in Ethiopia [15, 19, 31]. This could be due to healthcare providers working in PPM facilities having had more opportunities to receive updated training and supplies from the public health sector. However, due to the reservation of the public sector to provide primaquine to private-for-profit health facilities, these institutions found it a challenge to adhere to first line treatment recommendations of the guidelines for patients with *P. falciparum* or mixed infections. In addition, the qualitative data revealed that private healthcare providers prescribed antibiotics as per knowledge acquired through reading WHOs malaria case management guidelines. This is indicative of gaps in knowledge and lack of information on the importance of contextual guideline implementation to be effective and efficient in programme execution.

Single low dose primaquine is added with AL to treat *P. falciparum* or mixed infections to block onwards disease transmission [7, 32, 33]. No single *P. falciparum* or mixed infection cases were treated with the recommended artemisinin-based combination therapy plus single low dose (sld) primaquine. In addition, unlike the national recommendations, there are several cases that were treated with artemether monotherapy, chloroquine plus antibiotics, and only chloroquine for presumed cases. Therefore, the overall adherence of private healthcare providers to national malaria diagnosis and treatment guidelines was found to be suboptimal. This finding is in line with the findings of Bamiselu et al. [34], who reported adherence of 27.3% by private healthcare workers in Nigeria. This was reflected in clinical records that revealed there was a presumed malaria diagnosis after declaring a negative test result. This sub-optimal adherence could be explained due to the limited focus of the public health sector on formal private health facilities, in the management of malaria cases.

Though the private health sector in Ethiopia served more than one-third of malaria cases, only less than one-fifth of cases received standard recommended treatments in line with the national guidelines. This study highlights lack of orientation for private health sectors on the national guidelines and shortage as well as frequent stockout of nationally recommended anti-malarial supplies impede adherence with the guidelines. This study documented several challenges of the private health sector and their implications on malaria control and elimination programme in West Gojjam Zone and Ethiopia. However, many countries provide training, financial incentives, community awareness and develop clear governance in engaging the private health sector in malaria case management, referral, reporting, and surveillance activities [4, 35].

## Strengths and limitations

This study has both strengths and limitations. The results of the quantitative and qualitative inquiries adopted in this study helped investigators to triangulate the findings using several data sources. Hence, the study determines and explains factors for adherence of private healthcare providers to national malaria diagnosis and treatment guidelines. Ethiopia’s malaria control and elimination programme works to implement adherence to national diagnosis and treatment guidelines in order to halt disease transmission and prevent occurrence of anti-malarial drug resistance. The findings of the study show improvements in engaging formal private health facilities in malaria control and elimination activities.

Nonetheless, this study had some limitations. The first limitation is that the study only targeted adult uncomplicated malaria outpatient service beneficiaries. The estimated adherence of healthcare providers with national malaria diagnosis and treatment guidelines might be different with the inclusion of children, and severe and complicated cases. In addition, the accuracy of malaria diagnosis proficiency was not monitored through an External Quality Assurance (EQA) activity, which might have produced significant differences in the magnitude and species categorization. The second limitation of the study is it lacks the inclusion of comparison groups with public healthcare providers. The third limitation is related to the issue of data completeness during extraction of patient medical records. Therefore, it might be helpful to plan for a prospective randomized clinical trial to accurately estimate the magnitude of adherence of healthcare providers to national malaria guidelines and its associated predictors in the study areas.

## Conclusions

This sequential explanatory study determined an exemplary level of adherence of healthcare providers to parasitological confirmation. However, the private healthcare providers’ adherence to treatment guidelines was sub-optimal i.e., 20.0%. Countries like Ethiopia that have pledged to be effective in malaria control programmes and are heading towards the successful elimination of malaria from their territories by 2030 should work closely in malaria case management with private-for-profit health facilities. The determinants of suboptimal adherence of private healthcare providers to malaria diagnosis and treatment guidelines were unreliable laboratory supplies, interruption of anti-malarial drugs, and poor knowledge of the national standards. Malaria control and elimination efforts across Ethiopia could be improved through a win–win partnership model. In addition, the private health sector should share burden and use the information to improve adherence to malaria diagnosis, treatment, and reporting standards in the era of elimination. Therefore, the investigators recommend the Ministry of Health’s engagement in capacity enhancement of healthcare providers working in private-for-profit health facilities and ensuring the availability of uninterrupted anti-malarial drugs and laboratory supplies. Furthermore, it is recommended that prospective randomized studies be conducted, targeting the public health sector, private-for-profit, and private-for-non-profit health facilities.

## Supplementary Information

**Additional file 1: **Malaria diagnosis and treatment guidelines. depicts the malaria diagnosis and treatment guidelines recommendations. The criteria guide malaria case management for adult uncomplicated outpatients.

## Data Availability

The datasets used and/or analyzed during the current study are available from the corresponding author on reasonable request.

## Abbreviations

AL: Artemether-Lumefantrine
CI: Confidence Interval
DOT: Direct Observed Therapy
EFMOH: Ethiopian Federal Ministry of Health
EMIS: Ethiopia Malaria Indicator Survey
G6PDd: Glucose-6- Phosphate Dehydrogenase Deficiency
IDIs: In Depth Interviews
NGO: Non-Governmental Organization
NPPM: Non-Public–Private Mix
MDGs: Millennium Development Goals
PHCU: Primary Health Care Unit
PPM: Public -Private Mix
PPPs: Public–Private Partnerships
RDT: Rapid Diagnostic Test
RHB: Regional Health Bureau
SD: Standard Deviation
SSA: Sub-Sahara Africa
UNISA: University of South Africa
WHO: World Health Organization
