# Supplementary material for: Adherence of healthcare providers to malaria case management guidelines of the formal private sector in north-western Ethiopia: an implication for malaria control and elimination
Source: Malar J. 2022 Nov 21;21:347. doi: 10.1186/s12936-022-04379-0 (PMC9682744; doi:10.1186/s12936-022-04379-0)
Supplement: Supplementary file 1 — Additional file 1: Malaria diagnosis and treatment guidelines. depicts the malaria diagnosis and treatment guidelines recommendations. The criteria guide malaria case management for adult uncomplicated outpatients. [file 12936_2022_4379_MOESM1_ESM.pptx]

## Slide 1
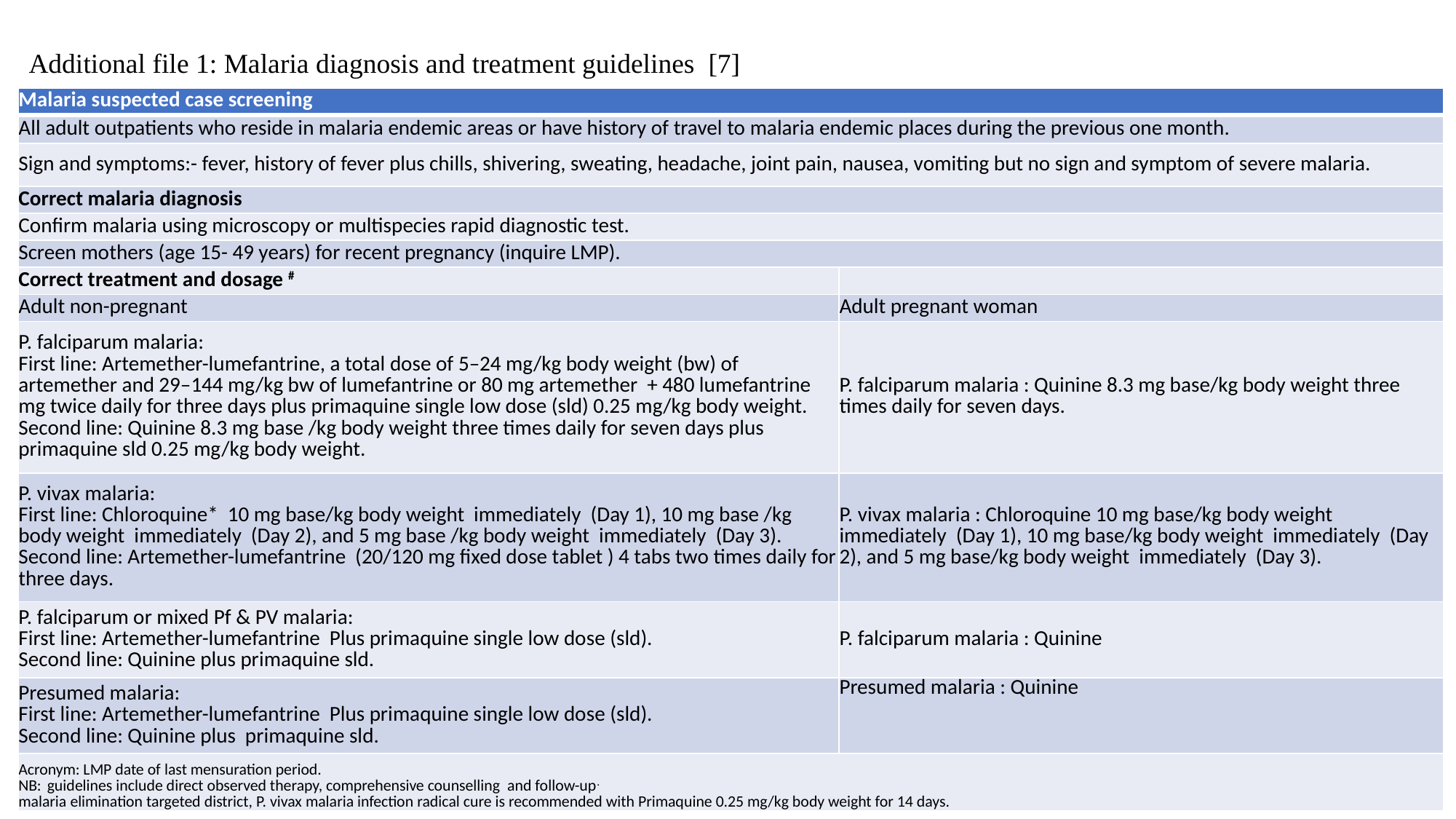

Additional file 1: Malaria diagnosis and treatment guidelines  [7]
| Malaria suspected case screening | |
| --- | --- |
| All adult outpatients who reside in malaria endemic areas or have history of travel to malaria endemic places during the previous one month. | |
| Sign and symptoms:- fever, history of fever plus chills, shivering, sweating, headache, joint pain, nausea, vomiting but no sign and symptom of severe malaria. | |
| Correct malaria diagnosis | |
| Confirm malaria using microscopy or multispecies rapid diagnostic test. | |
| Screen mothers (age 15- 49 years) for recent pregnancy (inquire LMP). | |
| Correct treatment and dosage # | |
| Adult non-pregnant | Adult pregnant woman |
| P. falciparum malaria: First line: Artemether-lumefantrine, a total dose of 5–24 mg/kg body weight (bw) of artemether and 29–144 mg/kg bw of lumefantrine or 80 mg artemether + 480 lumefantrine mg twice daily for three days plus primaquine single low dose (sld) 0.25 mg/kg body weight. Second line: Quinine 8.3 mg base /kg body weight three times daily for seven days plus primaquine sld 0.25 mg/kg body weight. | P. falciparum malaria : Quinine 8.3 mg base/kg body weight three times daily for seven days. |
| P. vivax malaria: First line: Chloroquine\* 10 mg base/kg body weight immediately (Day 1), 10 mg base /kg body weight immediately (Day 2), and 5 mg base /kg body weight immediately (Day 3). Second line: Artemether-lumefantrine (20/120 mg fixed dose tablet ) 4 tabs two times daily for three days. | P. vivax malaria : Chloroquine 10 mg base/kg body weight immediately (Day 1), 10 mg base/kg body weight immediately (Day 2), and 5 mg base/kg body weight immediately (Day 3). |
| P. falciparum or mixed Pf & PV malaria: First line: Artemether-lumefantrine Plus primaquine single low dose (sld). Second line: Quinine plus primaquine sld. | P. falciparum malaria : Quinine |
| Presumed malaria: First line: Artemether-lumefantrine Plus primaquine single low dose (sld). Second line: Quinine plus primaquine sld. | Presumed malaria : Quinine |
| Acronym: LMP date of last mensuration period. NB: guidelines include direct observed therapy, comprehensive counselling and follow-up. malaria elimination targeted district, P. vivax malaria infection radical cure is recommended with Primaquine 0.25 mg/kg body weight for 14 days. | |
